# Chitinase-like protein YKL-40 correlates with inflammatory phenotypes, anti-asthma responsiveness and future exacerbations

**DOI:** 10.1186/s12931-019-1051-9

**Published:** 2019-05-22

**Authors:** Lei Liu, Xin Zhang, Ying Liu, Li Zhang, Jing Zheng, Ji Wang, Philip M. Hansbro, Lei Wang, Gang Wang, Alan Chen-Yu Hsu

**Affiliations:** 1 Pneumology Group, Department of Integrated Traditional Chinese and Western Medicine, State Key Laboratory of Biotherapy, West China Hospital, Sichuan University, and Collaborative Innovation Centre for Biotherapy, Chengdu, 610041 Sichuan China; 2 Pneumology Group, Department of Integrated Traditional Chinese and Western Medicine, West China Hospital, Sichuan University, Chengdu, 610041 Sichuan China; 3 Johns Hopkins Asthma and Allergy Center, Johns Hopkins University School of Medicine, Baltimore, 21224 MD USA; 4 Department of Respiratory and Critical Care Medicine, Clinical Research Center for Respiratory Disease, West China Hospital, Sichuan University, Chengdu, 610041 Sichuan China; 5 Department of Integrated Traditional Chinese and Western Medicine, Xinqiao Hospital, Third Military Medical University, Chongqing, 400037 People’s Republic of China; 6 Centre for Inflammation, Centenary Institute, Sydney, NSW 2050 Australia; 7 Faculty of Science, University of Technology Sydney, Ultimo, NSW 2007 Australia; 8 Priority Research Centre for Healthy Lungs, Hunter Medical Research Institute, The University of Newcastle, New Lambton Heights, NSW 2305 Australia

**Keywords:** Inflammatory phenotypes, Asthma treatment response, Exacerbations, YKL-40

## Abstract

**Background:**

Asthma is a heterogeneous chronic airway disease, which may be classified into different phenotypes. YKL-40 is a chitin-binding glycoprotein with unclear functions, but its expression is associated with inflammation and tissue remodeling. However, few studies have explored whether YKL-40 is associated with inflammatory phenotypes of asthma.

**Methods:**

The study had two parts. Study I (*n* = 115) was a one-year prospective cohort designed to explore the relationship of serum YKL-40 levels with inflammatory phenotypes of asthma at baseline, and during exacerbations. Study II (*n* = 62) was a four-week prospective cohort designed to define whether serum YKL-40 levels could predict responses to a fixed anti-asthma regimen. YKL-40, IL-6 and CCL22 levels were detected using ELISA, and a sputum inflammatory panel (including IL-1β, IL-5, IL-8 and TNF-α) was assessed using Luminex-based MILLIPLEX assay.

**Results:**

Study I: Serum YKL-40 levels in non-eosinophilic asthma (NEA) i.e. neutrophilic (47.77 [29.59, 74.97] ng/mL, *n* = 40) and paucigranulocytic (47.36 [28.81, 61.68] ng/mL, *n* = 31) were significantly elevated compared with eosinophilic asthma (31.05 [22.41, 51.10] ng/mL, *n* = 44) (*P* = 0.015). Serum YKL-40levels positively correlated with blood neutrophils, sputum IL-1β and plasma IL-6 but negatively correlated with serum IgE and blood eosinophils (all *P* ≤ 0.05). Baseline YKL-40 levels predicted moderate to severe exacerbations within a one-year period (aOR = 4.13, 95% CI = [1.08, 15.83]). Study II: Serum YKL-40 was an independent biomarker of negative responses to anti-asthma regimens (adjusted Odds Ratio [aOR] = 0.82, 95% CI = [0.71, 0.96].

**Conclusions:**

These studies show that YKL-40 is a non-type 2 inflammatory signature for NEA, which could predict responsiveness or insensitivity to anti-asthma medications and more exacerbations. Further studies are needed to assess whether monitoring YKL-40 levels could provide potential implications for clinical relevance.

**Electronic supplementary material:**

The online version of this article (10.1186/s12931-019-1051-9) contains supplementary material, which is available to authorized users.

## Background

Asthma is an inflammatory disease characterized by airway hyper-responsiveness and remodeling [1], and may be classified into different phenotypes [2, 3]. Indeed, sputum granulocyte levels [4] can be used to define asthma as eosinophilic, neutrophilic, paucigranulocytic or mixed granulocytic asthma. Assessment of airway inflammation in asthma is becoming increasingly important, as the inflammatory phenotype underpins the treatment response, with the non-eosinophilic form of the disease responding poorly to the inhaled corticosteroid (ICS) therapy [5]. Molecular-targeted treatments or biomarkers are being actively investigated for neutrophilic asthma [6]. Potential biomarkers that are reproducible, non-invasive and uninfluenced by treatments need to be identified, and significant efforts are required to find those that can be used to indicate personalized treatment based on asthma phenotypes and endotypes [7]. This is currently a major priority in asthma research [8].

A group of proteins recently discovered to be potential biomarkers of asthma are the chitinases and chitinase-like proteins. Two members of this family, the enzymatically active chitotriosidase and the enzymatically inactive chitinase-like protein YKL-40, may play important roles in driving asthma disease pathogenesis. Circulating levels of YKL-40 have been shown to be elevated in a variety of diseases including metastatic breast cancer, hepatic fibrosis, severe purulent meningitis and community acquired pneumonia [9–12]. Increased levels of YKL-40 have also been implicated in rheumatoid arthritis, atherosclerosis and osteoarthritis where their expression correlated with disease activity [13–15]. Moreover, serum YKL-40 levels are increased in asthmatic patients and may be involved in airway remodeling [16, 17] . Circulating YKL-40 levels correlated with asthma severity, thickness of the subepithelial basement membrane, exacerbations and pulmonary function, which indicates that circulating YKL-40 levels are a promising biomarker for asthma [18–20]. In addition, blood eosinophils, the fraction of exhaled nitric oxide (F_E_NO), serum periostin, and serum immunoglobulin E (IgE) are indicative biomarkers of eosinophilic asthma (EA) in clinical research and practice [21]. Interestingly, a few studies have found negative correlations between YKL-40 and these biomarkers [1, 20, 22]. Thus, it remains unclear if YKL-40 is differentially expressed in eosinophilic and non-eosinophilic asthma (EA and NEA). We hypothesized YKL-40 would be a specific biomarker for NEA.

There are phenotypes of asthmatic patients with type 2 (T2)-low inflammation [2], characterized by low eosinophilia, and high neutrophilic (sputum neutrophils > 40–60%) or paucigranulocytic (i.e., normal sputum levels of eosinophils and neutrophils) inflammation [23]. However, our studies found that much remains need to be understood about these patients and the underlying causes of their disease [23, 24]. These individuals can exhibit highly variable inflammatory and physiological profiles and often respond poorly to corticosteroids [8, 25]. Dissection of the inflammatory pattern of these patients has revealed significantly higher levels of pro-inflammatory cytokines, such as interleukin (IL)-6 and IL-8, which are associated with airway neutrophilia [26, 27], and our study found that activation of the NLRP3 inflammasome and elevated IL-1β production [28]. A recent study by Gomez et al., suggested that YKL-40 may be a non-type 2 (T2) rather than T2 biomarker [20]. Nevertheless, few studies have explored the relationships between serum YKL-40, and non-T2 inflammation, responsiveness to therapy, or exacerbation.

Thus, this study was designed to investigate whether: (1) increased serum YKL-40 levels are associated with NEA phenotypes; (2) YKL-40 could be indicative of response to treatment; and (3) YKL-40 indicates the risk of asthma exacerbation in a real-world setting.

## Methods

### Design overview and subjects

This study consisted of two parts, which were based on the Severe Asthma Web-based Database (SAWD) from the Australasian Severe Asthma Network (ASAN) in a real-world setting [29]. All patients with asthma received treatments that were determined by their treating physicians’ standard practices. Study I was a one-year prospective cohort study designed to explore the relationship of serum YKL-40 levels with inflammatory phenotypes of asthma at baseline and during exacerbations in the following 12 months. Study II was a four-week prospective cohort study to define whether serum YKL-40 levels would predict response to a fixed asthma treatment regimen. The institutional review board (IRB) at West China Hospital, Sichuan University (Chengdu, China) reviewed and approved this study (No. 2014–30). This cohort study was registered with ChiCTR-OOC-16009529 at http://www.chictr.org.cn.

In study I, subjects aged ≥18 years old with stable asthma (*n* = 115) were recruited from the Asthma Clinic of West China Hospital at Sichuan University, China. Asthma was diagnosed according to American Thoracic Society (ATS) [30] and Global Initiative for Asthma (GINA) guidelines [31] based on current (within the previous 12 months) episodic respiratory symptoms, and confirmed by evidence of either airway hyper-responsiveness or bronchodilator responsiveness with improvement of FEV_1_ ≥ 12% and 200 mL after 400 μg of salbuterol (GSK, Avda de Extremadura, Spain) delivered by metered-dose inhaler and spacer (150 mL, Vanbo Technology Corp. Shanghai, China). Subjects with recent (past month) respiratory tract infection, rheumatoid arthritis, or other severe unstable chronic diseases were excluded. We also excluded those who were pregnant or breast-feeding at the time of recruitment and those who were unable to perform the required measurements. In study II, subjects (*n* = 62) were administered by 4-week fixed treatment and then the association between responsiveness to treatment and YKL-40 levels was assessed. All subjects gave written informed consents prior to participation.

### Data collection and clinical assessments

Baseline clinical data from subjects with stable asthma were collected. These data included socio-demographics, medications, atopy, asthma history, fractional exhaled nitric oxide (F_E_NO), asthma control questionnaire (ACQ), and asthma quality of life questionnaire (AQLQ) data, which have been validated for the Chinese population [32–34]. All subjects underwent sputum induction and blood sampling in the morning of study entry.

### Sputum induction, analysis and cytokines detection

Sputum induction and analysis were performed as described previously [35]. Briefly, sputum was induced after pre-treatment with 400 μg salbutamol using 4.5% saline atomized with an ultrasonic nebulizer (Cumulus, HEYER Medical AG, German). If pre- or post-FEV_1_ was less than 40% predicted, sputum was induced with 0.9% saline after it was deemed safe by the supervising physician. For inflammatory cell counts, selected sputum plugs were dispersed using dithiothreitol (DTT), and a total cell count and viability was performed. Cytospins were prepared using centrifugation-smear (CYTOPRO 7620, WESCOR®, INC, LOGAN, USA) and stained (May-Grunwald Giemsa), and a differential cell count was obtained from 40 non-squamous cells. Differential cell counts were performed by well-trained researchers from Australia and China. The supernatant from induced sputum samples was aspirated and frozen at − 80 °C until assessment.

Inflammatory mediators and cytokines in sputum supernatant, including IL-1β, IL-5, IL-8 and tumor necrosis factor (TNF)-α were assessed using Luminex-based MILLIPLEX® MAP Human Cytokine/Chemokine Magnetic bead Panel Kit (EMD Millipore Corporation, Billerica, MA, USA), and analysed using Milliplex Analyst 5.1 software. The minimum detectable concentrations of the cytokines in sputum supernatant were 0.8 pg/mL, 0.5 pg/mL, 0.4 pg/mL and 0.7 pg/mL, respectively. Spiking experiments of cytokines in sputum supernatants showed that recovery ranged from 70 to 130% for all detectable analyses [36, 37].

### Definition of airway cellular inflammatory phenotypes

Sputum cellular phenotypes were classified as EA (eosinophils ≥3%), and NEA [38], which included neutrophilic (NA, neutrophils ≥61% and eosinophils < 3%), and paucigranulocytic (PGA, neutrophils < 61% and eosinophils < 3%) asthma based on the presence of sputum granulocytes [4].

### Blood analyte assays

Venous blood samples that require fasting on the study entry were collected either in ethylenediamine tetra acetic acid treated tubes for total and differential blood cell counts or untreated tubes to obtain serum for determining YKL-40 and IgE assays. Serum samples were stored at − 80 °C until analysis. Total and differential blood cell counts were obtained (Sysmex XN-9000 hematology analyser, Sysmex Corporation, Kobe, Japan). Cells were classified as neutrophils, eosinophils or monocytes according to standard morphological criteria, and absolute numbers of each cell type were calculated. Serum YKL-40, plasma IL-6 and CCL22 levels were determined by ELISA (R&D Systems, Minneapolis, Minnesota, USA). The minimum detectable dose of these cytokines were 8.15 pg/mL, 0.7 pg/mL and 62.5 pg/mL. In addition, serum IgE levels were measured by immunoassay (Beckman Immage 800 immunoassay analyser, Beckman Coulter Inc., USA), with a minimum detectable level of IgE of 5.0 IU/mL.

### Asthma exacerbation

In Study I, in the one-year prospective cohort study, 115 patients were classified to serum YKL-40^high/low^ groups at baseline according to median levels. All subjects were followed up with a 12-month period (face-to-face visits or telephone calls if unavailable) and assessed exacerbations. Asthma outcomes included moderate to severe exacerbation leading to non-planned visit, emergency department (ED) visit, and hospitalization. Detailed definitions were based on the criterion of the ATS/ European Respiratory Society (ERS) Task Force [39]. Researchers who evaluated asthma exacerbation were blinded to the results of serum YKL-40 levels.

### Assessment of asthma treatment responsiveness

In study II, asthmatics (*n* = 62) were divided into the EA and NEA groups, who completed the 4-week fixed therapy with ICS plus long-acting beta-agonist (LABA). Those subjects were assessed at baseline and after treatments, and were excluded from the analysis if they changed their medications within this period. ACQ and spirometry were carried out to define treatment responder as one or more of the following: ≥ 0.5 point decrease in ACQ or ≥ 12% increase of FEV_1_ [35]. Researchers who assessed treatment responsiveness were blinded to the results of serum YKL-40 and inflammatory phenotype assessment.

### Statistical analysis

Continuous variables are presented as mean ± standard deviation (SD) or median (Quartiles [Q] 1, 3) depending on their distribution assessed by Kolmogorov-Smirnov test. Percentages are shown for categorical data such as gender. If possible, all data were transformed to normal distribution. For parametric data, two or more groups were compared using the Student’s *t*-test or one-way ANOVA, respectively. For non-parametric data, two or more groups were compared using the Mann-Whitney or Kruskal-Wallis tests, respectively. Covariance analysis was constructed to adjust for differences in baseline characteristics. Categorical data were compared using a Chi-square test or Fisher’s exact test. Correlations were assessed using Pearson’s or Spearman’s coefficient tests. We used logistic regression models to evaluate the relationships between serum YKL-40, treatment response and exacerbations. Adjusted odds ratio (aOR) with 95% confidence interval (CI) were calculated. Statistical analysis was performed with the IBM SPSS 21.0 software (SPSS, Chicago, IL, USA). All tests were two-tailed, and a *P*-value ≤0.05 was considered statistically significant.

## Results

### Characteristics of subjects grouped by airway inflammatory phenotypes

As there were only two participants with a mixed granulocytic inflammatory phenotype (sputum neutrophils ≥61% and sputum eosinophils ≥3%) in study I, they were not included in the analysis. Accordingly, the subjects included with stable asthma were divided into the EA (*n* = 44), NA (*n* = 40) and PGA (*n* = 31) groups. Compared with the EA subjects, the NA group was older (41.6 ± 12.5 vs. 49.4 ± 15.6 yr., *P =* 0.014), had longer asthma duration (3.00 [1.25, 13.50] vs. 13.00 [4.50, 28.75] yr., *P =* 0.002), but had lower F_E_NO levels (53.0 [37.0, 80.0] vs. 21.0 [15.0, 33.0] ppb, *P* < 0.001). No statistical differences were observed in gender, BMI, smoking, asthma onset, medications/ doses, FEV_1_% predicted, FEV_1_/FVC, ACQ, AQLQ across three groups (all *P >* 0.05) (Table 1). Subjects with PGA had lower IgE levels than those with EA and NA (58.7 [27.5, 215.0] vs. 258.3 [170.6, 537.5] vs. 108.6 [29.4, 403.2] IU/mL, *P* < 0.001).

**Table 1:** Study I: Characteristics of asthma patients grouped by EA and NEA

Characteristics	EA	NEA			t_1_/χ^2^_1_/z_1_	*P*_1_	F_2_/χ^2^_2_/z_2_	*P*_2_
		Total	NA	PGA				
n	44	71	40	31				
Age, mean ± SD, yr	41.6 ± 12.5	48.6 ± 15.3	49.4 ± 15.6^*^	47.6 ± 15.2	−2.557	0.012	3.389	0.037
Gender, male n (%)	15 (34.1)	33 (46.5)	20 (50.0)	13 (41.9)	1.714	0.190	2.181	0.336
BMI, median (Q1, Q3), kg/m^2^	22.78 ± 4.11	24.04 ± 4.36	24.11 ± 4.65	23.96 ± 4.02	−1.539	0.127	1.185	0.310
Smoking, Current/Ex/Non	5/5/34	9/16/46	6/9/25	3/7/21	2.504	0.286	2.979	0.561
Smoking, pack-years	0.00 (0.00,0.00)	0.00 (0.00,7.70)	0.00 (0.00,7.70)	0.00 (0.00,8.80)	3.390	0.184	3.390	0.184
Age of asthma onset, mean ± SD, yr	33.73 ± 14.74	34.38 ± 19.18	32.65 ± 18.95	36.61 ± 19.54	−0.193	0.847	0.460	0.633
Asthma duration, median (Q1, Q3), yr	3.00 (1.25, 13.50)	7.00 (3.00, 23.00)	13.00 (4.50, 28.75)^*^	6.00 (2.00, 15.00)	−2.763	0.006	10.187	0.006
ICS dose, BDP equivalent, median (Q1, Q3), μg/d	400 (400, 400)	400 (400, 400)	400 (400, 400)	400 (400, 800)	−0.261	0.794	2.698	0.260
GINA steps 1–5, (n)	0/0/40/4/0	0/0/65/6/0	0/0/39/1/0	0/0/26/5	0.014	0.906	4.013	0.144
Medications, n (%)								
ICS/LABA	44 (100.0)	70 (98.6)	40 (100.0)	30 (96.8)	0.625	1.000	2.291	0.270
LTRA	40 (90.9)	62 (87.3)	34 (85.0)	28 (90.3)	0.348	0.774	0.838	0.753
Theophyline	7 (15.9)	13 (18.3)	7 (17.5)	6 (19.4)	0.109	0.741	0.151	0.952
SABA	2 (4.5)	1 (1.4)	1 (2.5)	1 (3.2)	0.625	1.000	0.498	1.000
Spirometry, mean ± SD								
FEV_1_, L	2.27 ± 0.86	2.23 ± 0.93	2.12 ± 0.85	2.31 ± 0.99	0.233	0.816	0.261	0.771
FVC, L	3.24 ± 0.96	3.18 ± 0.88	3.15 ± 0.84	3.21 ± 0.95	0.333	0.740	0.095	0.910
FEV_1_, % predicted	73.51 ± 19.60	75.37 ± 20.46	72.76 ± 21.79	78.65 ± 18.47	−0.478	0.634	0.856	0.428
FVC, % predicted	88.02 ± 13.92	90.80 ± 14.27	89.69 ± 15.31	92.19 ± 12.96	−1.021	0.309	0.788	0.457
FEV_1_/FVC, %	69.40 ± 14.10	68.08 ± 15.26	66.20 ± 15.88	70.46 ± 14.36	0.460	0.647	0.823	0.442
ACQ scores, median (Q1, Q3)	1.0 (0.17, 1.63)	0.5 (0.0, 1.33)	0.5 (0.0, 1.33)	0.5 (0.0, 1.00)	2.259	0.109	2.259	0.109
AQLQ scores, mean ± SD	5.75 ± 0.80	5.99 ± 0.64	5.94 ± 0.64	6.07 ± 0.64	−1.765	0.080	1.835	0.164
F_E_NO, median (Q1, Q3), ppb	53.0 (37.0, 80.0)	21.0 (15.0,32.0)	21.0 (15.0,33.0)^*^	21.5 (14.5, 30.25)^*^	5.444	< 0.001	6.544	0.002
IgE, median (Q1, Q3), IU/mL	258.3 (170.6, 537.5)	73.07 (28.66, 271.17)	108.6 (29.4, 403.2)	58.7 (27.5, 215.0)^*^	3.891	< 0.001	8.593	< 0.001

*ACQ* asthma control questionnaire, *AQLQ* asthma quality of life questionnaire, *BDP* beclomethasone equivalents, *BMI* body mass index, *F*_*E*_*NO* fractional exhaled nitric oxide, *FEV*_*1*_ forced expiratory volume in 1 s, *FVC* forced vital capacity, *GINA* Global Initiative for Asthma, *ICS* inhaled corticosteroid, *ICS/LABA* inhaled corticosteroid with long-acting beta-agonist, *LTRA* leukotriene receptor antagonist, *SABA* short-acting beta-agonist, *SD* standard deviation, *Q* quartile, *EA* eosinophilic asthma, *NEA* non-eosinophilic asthma

^*^Compared with eosinophilic-asthma, *P* < 0.05. t_1_ /χ^2^_1_/z_1_ and *P*_1_: Compared between EA and NEA. F_2_/χ^2^_2_/z_2_ and *P*_2_: Compared across EA, NA and PGA

### Airway and systemic inflammation

Compared with the EA subjects, the NA group had higher levels of blood neutrophils (56.50 ± 7.67 vs. 61.96 ± 8.76, *P* = 0.003 for percentage), plasma CCL22 (600.7 [398.8, 968.2] vs. 920.6 [604.6, 1108.5] pg/mL, *P* = 0.029), sputum neutrophils (0.88 [0.36, 3.32] vs. 5.93 [2.57, 11.70] × 10^6^/L, *P* < 0.001 for absolute numbers, and 42.50 [15.50, 57.75] vs. 87.25 [70.25, 96.00] %, *P* = 0.029 for percentage), sputum IL-1β (11.90 [5.77, 28.71] vs. 67.09 [8.64, 476.99] pg/mL, *P* < 0.001), IL-8 (1272.00 [638.28, 2047.75] vs. 2218.00 [1014.00, 3702.00] pg/mL, *P* = 0.009) and TNF-α (10.10 [4.66, 20.19] vs. 34.02 [8.64, 63.80] pg/mL, *P* = 0.001), but lower eosinophils in blood and sputum (all *P >* 0.05) (Table 2). There were no statistically significant differences in plasma IL-6 and sputum IL-5 across the three groups. Furthermore, compared with the EA subjects, the subjects with PGA had higher levels of blood neutrophils (56.50 ± 7.67 vs. 64.42 ± 8.49, *P* < 0.001 for percentage) and plasma CCL22 (600.7 [398.8, 968.2] vs. 871.7 [504.7, 1511.4] pg/mL, *P* = 0.033), but lower eosinophils in blood and sputum. Compared with the NA subjects, the subjects with PGA had higher sputum macrophages (0.68 [0.33, 1.46] vs. 1.55 [1.10, 3.23] × 10^6^/L, *P* = 0.003 for absolute numbers, and 11.25 [4.00, 29.25] vs. 71.38 [50.75, 87.13] %, *P* < 0.001 for percentage).

**Table 2:** Study I: Airway and systemic inflammation in asthma patients grouped by EA and NEA

Variables	EA	NEA			t_1_/χ^2^_1_/z_1_	*P*_1_	F_2_/χ^2^_2_/z_2_	*P*_2_
		Total	NA	PGA				
Blood								
Eosinophils, 10^9^/L	0.43 (0.33, 0.59)	0.14 (0.09, 0.19)	0.14 (0.10, 0.17) ^*^	0.13 (0.07, 0.20) ^*^	−8.718	< 0.001	39.043	< 0.001
Eosinophils, %	6.21 (5.07, 9.12)	2.13 (1.56, 2.93)	2.24 (1.71, 2.87) ^*^	1.81 (0.97, 3.29) ^*^	−8.350	< 0.001	34.672	< 0.001
Neutrophils, 10^9^/L	3.01 (2.96, 4.14)	3.85 (2.95, 5.02)	3.84 (2.74, 5.00)	3.88 (3.32, 5.02)	−0.522	0.602	1.043	0.356
Neutrophils, %	56.50 ± 7.67	63.00 ± 8.67	61.96 ± 8.76^*^	64.42 ± 8.49^*^	−4.059	< 0.001	8.494	< 0.001
Monocytes, 10^9^/L	0.38 ± 0.13	0.39 ± 0.15	0.40 ± 0.13	0.39 ± 0.17	− 0.605	0.546	0.184	0.832
Monocytes, %	5.48 (4.74, 6.39)	6.47 (4.96, 7.31)	6.56 (5.12, 7.68) ^*^	5.62 (4.31, 7.03)	−2.000	0.048	3.710	0.028
YKL-40, ng/mL	31.05 (22.41, 51.10)	47.36 (29.47, 69.80)	47.77 (29.59, 74.97) ^*^	47.36 (28.81, 61.68) ^*^	−2.976	0.004	4.388	0.015
CCL22, pg/mL	600.7 (398.8, 968.2)	920.6 (600.7, 1174.7)	920.6 (604.6, 1108.5) ^*^	871.7 (504.7, 1511.4) ^*^	−2.591	0.011	3.315	0.042
IL-6, pg/mL	1.91 (1.16, 3.48)	1.91 (0.77, 3.72)	1.79 (0.81, 3.61)	2.05 (0.65, 3.89)	0.628	0.532	0.303	0.740
Induced sputum								
Eosinophils, 10^6^/mL	0.11 (0.00, 0.31)	0.00 (0.00, 0.01)	0.01 (0.00, 0.03) ^*^	0.00 (0.00, 0.01) ^*^	−3.003	0.003	10.701	0.005
Eosinophils, %	1.75 (0.00, 8.50)	0.00 (0.00, 0.25)	0.25 (0.00, 0.75) ^*^	0.00 (0.00, 0.25) ^*^	−3.284	0.001	11.986	0.002
Neutrophils, 10^6^/mL	0.88 (0.36, 3.32)	5.93 (2.57, 11.70)	5.93 (2.57, 11.70) ^*^	0.63 (0.22, 1.39)	−0.056	0.956	16.969	< 0.001
Neutrophils, %	42.50 (15.50, 57.75)	40.38 (16.25, 63.38)	87.25 (70.25, 96.00) ^*^	27.88 (12.56, 46.31) ^*^	0.124	0.902	23.306	< 0.001
Macrophages, 10^6^/mL	1.46 (0.62, 1.96)	1.40 (0.81, 2.91)	0.68 (0.33, 1.46)	1.55 (1.10, 3.23) ^#^	−0.596	0.551	8.634	0.013
Macrophages, %	55.00 (27.25, 74.50)	60.00 (35.75, 82.25)	11.25 (4.00, 29.25)	71.38 (50.75, 87.13) ^#^	−0.731	0.465	24.660	< 0.001
IL-1β, pg/mL	11.90 (5.77, 28.71)	22.19 (8.77, 90.90)	67.09 (8.64, 476.99) ^*^	16.52 (8.84, 40.71)	−2.175	0.030	10.361	< 0.001
IL-5, pg/mL	1.91 (1.11, 6.04)	1.50 (0.95, 3.13)	1.12 (0.91, 3.50)	1.68 (1.05, 2.94)	−0.767	0.443	0.811	0.667
IL-8, pg/mL	1272.00 (638.28, 2047.75)	1950.00 (977.28, 3199.00)	2218.00 (1014.00, 3702.00) ^*^	1813.50 (895.27, 3106.00)	−2.395	0.017	3.818	0.026
TNF-α, pg/mL	10.10 (4.66, 20.19)	14.81 (7.49, 46.77)	34.02 (8.64, 63.80) ^*^	12.77 (7.00, 27.48)	−2.264	0.024	6.008	0.004

*CCL* chemokine (C-C motif) ligand, *EA* eosinophilic asthma, *GINA* Global Initiative for Asthma, *IL* interleukin, *NA* neutrophilic asthma, *NEA* non-eosinophilic asthma, *PGA* paucigranulocytic asthma

^*^Compared with eosinophilic asthma, *P* < 0.05. ^#^Compared with neutrophilic asthma, *P* < 0.05. t_1_/χ^2^_1_/z_1_ and *P*_1_: Compared between EA and NEA. F_2_/χ^2^_2_/z_2_ and *P*_2_: Compared across EA, NA and PGA

### YKL-40 levels in serum across inflammatory phenotypes

**Fig. 1:**
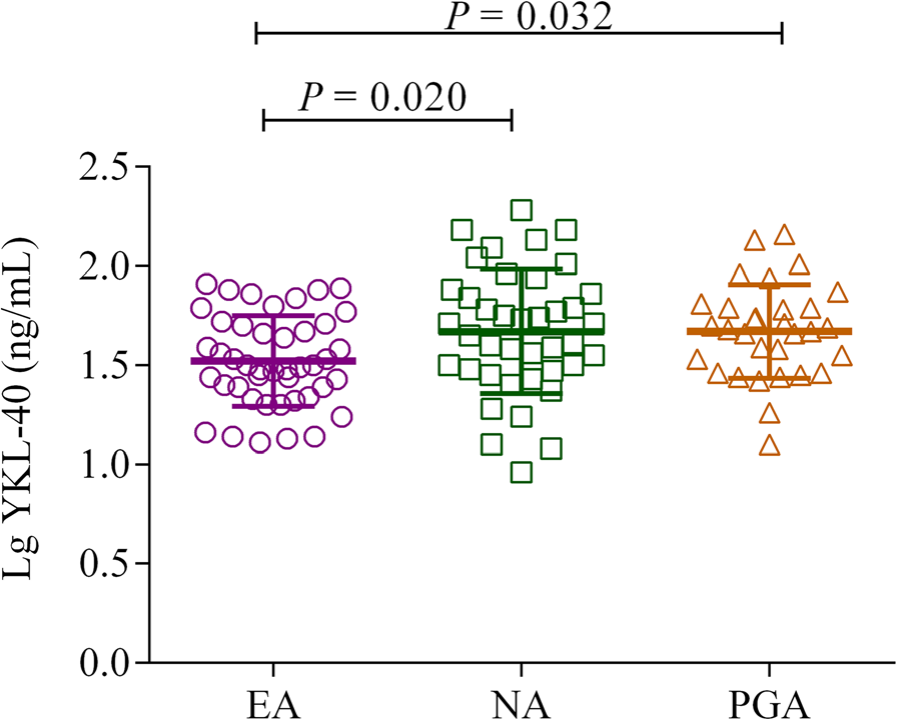
Serum levels of YKL-40 in patients with EA, NA and PGA phenotypes. EA = eosinophilic asthma; NA = neutrophilic asthma; PGA = paucigranulocytic asthma. *P* values are shown for comparisons with EA. Results are presented as individual data points with means and standard deviation bars

To determine if clinical and phenotypic differences correlate with YKL-40 expression, we measured its’ levels in the serum. Compared with EA subjects (31.05 [22.41, 51.10] ng/mL), patients with NEA, either NA (47.77 [29.59, 74.97] ng/mL) or PGA (47.36 [28.81, 61.68] ng/mL) had significantly elevated serum YKL-40 levels (*P* = 0.020 and *P* = 0.032, respectively) (Fig. 1). We assessed the effects of age on the relationship between YKL-40 and NEA, and covariance analysis showed that the statistical between-group difference in YKL-40 remained significant after adjustment for age (*P* = 0.044).

Correlation analyses showed that the serum YKL-40 levels were positively correlated with age (*r* = 0.55, *P* < 0.001) and smoking pack-years (*r* = 0.22, *P* = 0.019), but not ACQ scores (*r* = − 0.06, *P* = 0.559) or F_E_NO (*r* = − 0.12, *P* = 0.220) (Table 3). YKL-40 negatively correlated with IgE levels (*r* = − 0.22, *P* = 0.021) and airflow obstruction (*r* = − 0.27, *P* = 0.004 for FEV_1_% predicted, and *r* = − 0.34, *P* < 0.001 for FEV_1_/FVC %). We also found that the YKL-40 levels correlated with eosinophil (*r* = − 0.23, *P* = 0.016 for absolute numbers, and *r* = − 0.24, *P* = 0.009 for percentage), neutrophil (*r* = 0.09, *P* = 0.365 for absolute numbers, and *r* = 0.19, *P* = 0.046 for percentage) and monocyte (*r* = 0.23, *P* = 0.016 for absolute numbers, and *r* = 0.26, *P* = 0.005 for percentage) in blood but not any granulocytes in sputum (all *P* > 0.05). Plasma IL-6 (*r* = 0.32, *P =* 0.003) and sputum IL-1β (*r* = 0.24, *P =* 0.037), but not IL-5 (*r* = − 0.01, *P =* 0.907) or TNF-α (*r* = 0.10, *P =* 0.376) correlated with serum YKL-40. However, the relationships between plasma CCL22, sputum IL-8 and serum YKL-40 were not statistically significant (*r* = 0.21, *P =* 0.066 and *r* = 0.20, *P =* 0.077, respectively).

**Table 3:** Correlations between serum YKL-40 and clinical characteristics

Clinical characteristics	YKL-40 (ng/mL)	
	*r*	*P* value
Age, yr.^a^	0.55	< 0.001
Smoking, pack-years	0.22	0.019
FEV_1_% predicted^a^	−0.27	0.004
FEV_1_/FVC %^a^	−0.34	< 0.001
ACQ	−0.06	0.559
F_E_NO, ppb^a^	−0.12	0.220
IgE, IU/mL^a^	−0.22	0.021
Cell count in peripheral blood		
Eosinophils, 10^9^/L	−0.23	0.016
Eosinophils, %	−0.24	0.009
Neutrophils, 10^9^/L^a^	0.09	0.365
Neutrophils, %^a^	0.19	0.046
Monocytes, 10^9^/L^a^	0.23	0.016
Monocytes, %^a^	0.26	0.005
Systemic cytokines		
IL-6, pg/mL^a^	0.32	0.003
CCL22, pg/mL^a^	0.21	0.066
Cell count in induced sputum		
Eosinophils, 10^6^/mL	0.05	0.710
Eosinophils, %	0.02	0.859
Neutrophils, 10^6^/mL	−0.01	0.913
Neutrophils, %^a^	0.08	0.567
Macrophages,10^6^/mL	−0.18	0.175
Macrophages, %^a^	−0.07	0.622
Cytokines in sputum		
IL-1β, pg/mL	0.24	0.037
IL-5, pg/mL	−0.01	0.971
TNF-α, pg/mL^a^	0.10	0.376
IL-8, pg/mL	0.20	0.077

*ACQ* asthma control questionnaire, *CCL* chemokine (C-C motif) ligand, *F*_*E*_*NO* fractional exhaled nitric oxide, *FEV*_*1*_ forced expiratory volume in 1 s, *FVC* forced vital capacity, *IL* interleukin, *TNF* tumor necrosis factor

^a^Pearson’s coefficient tests

### Asthma exacerbation and multivariate logistic regression analyses

One hundred and nine patients who completed the 12-month follow-up were divided into the YKL-40^low^ (*n* = 57) and YKL-40^high^ (*n* = 52) groups. Twenty-one patients (19.3%) underwent moderate to severe exacerbations during follow-up. We showed that there was no difference in moderate to severe exacerbations between these two groups (all *P* > 0.05) during the 12-month follow-up (Additional file 1: Table S1).

**Fig. 2:**
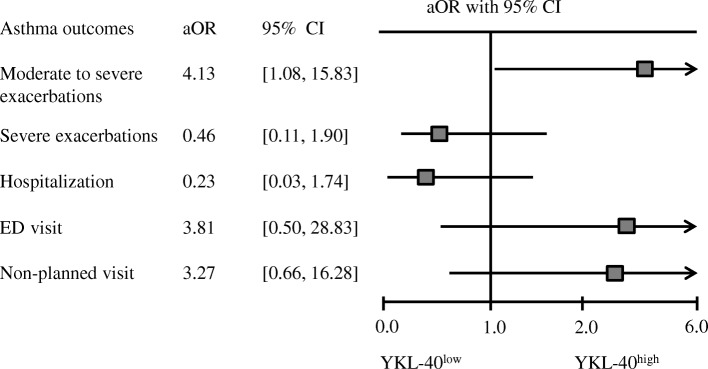
Forest plot of the potential of serum YKL-40 levels to predict exacerbations within the 12-month follow-up period. ED = emergency department; CI = confidence interval; aOR = adjusted odds ratio

To explore the relationship of YKL-40 with future risk of asthma exacerbation, we used multivariate logistic regression models to adjust for gender, age, BMI, smoking status, exacerbations over the previous year, FEV_1_%predicted and ICS dosage. Serum YKL-40 level was associated with an increased risk of moderate to severe exacerbation (aOR = 4.13, 95% CI = [1.08, 15.83]), but no differences were found in severe exacerbations (aOR = 0.46, 95% CI = [0.11, 1.90]), hospitalization (aOR = 0.23, 95% CI = [0.03, 1.74]), ED visit (aOR = 3.81, 95% CI = [0.50, 28.83]) and non-planned visit (aOR = 3.27, 95% CI = [0.66, 16.28]) (Fig. 2).

### Serum YKL-40 and asthma treatment responsiveness

**Fig. 3:**
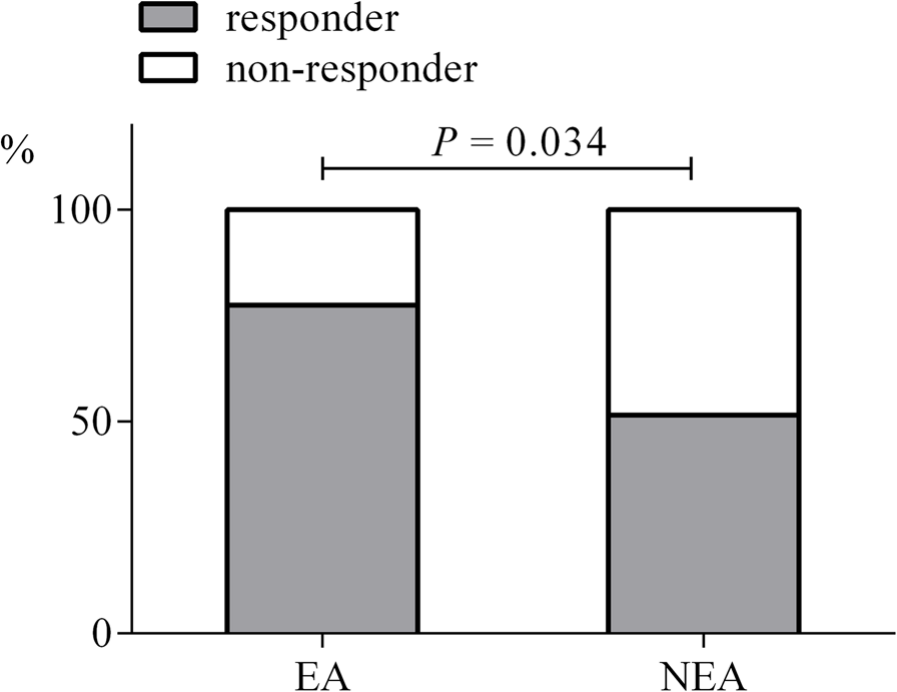
Treatment responder between EA and NEA groups. Responder was defined by ΔACQ ≥ 0.5 or ΔFEV_1_ ≥ 12%; non-responder was defined by ΔACQ < 0.5 and ΔFEV_1_ < 12%. EA = eosinophilic asthma; NEA = non-eosinophilic asthma

**Fig. 4:**
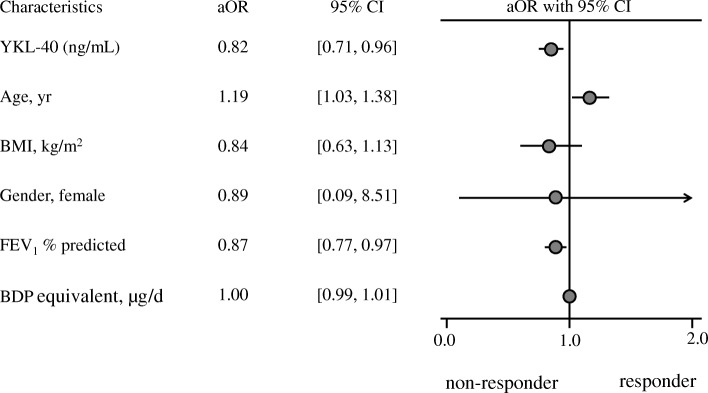
Forest plot of serum YKL-40 in predicting asthma treatment responsiveness. Responder was defined by ΔACQ ≥ 0.5 or ΔFEV_1_ ≥ 12%. non-responder was defined by ΔACQ < 0.5 and ΔFEV_1_ < 12%. ACQ = asthma control questionnaire; BDP = beclomethasone equivalents; BMI = body mass index; CI = confidence interval; FEV_1_ = forced expiratory volume in 1 s; aOR = adjusted odds ratio

In study II, the asthma subjects included were divided into the EA (*n* = 31) and non-EA (n = 31) groups. Compared with the EA subjects, the NEA group was older (40.42 ± 12.76 vs. 48.97 ± 16.23 yr., *P =* 0.025), but had lower F_E_NO (55.0 [47.5, 80.5] vs. 19.0 [16.0,48.50] ppb, *P =* 0.001) and IgE levels (258.70 [178.76, 605.03] vs. 71.57 [26.02, 444.70] IU/mL, *P =* 0.002) at baseline. The sociodemographic and clinical characteristics including serum YKL-40 levels between the two cohorts from studies I and II were comparable (all *P >* 0.05, Additional file 1: Tables S2 and S3). The subjects in the EA group had better improvement in ΔACQ (− 0.67 [− 1.5, − 0.17] vs. -0.17 [− 1.00, 0.17], *P =* 0.078) and ΔFEV_1_ (5.85 [− 4.77, 29.44] vs. -1.73 [− 9.27, 11.30] %, *P =* 0.198), but this did not reach statistical significance. However, based on the pre-specified definition either in ΔACQ or ΔFEV_1_, a greater proportion of clinically significant improvement in the EA group was achieved than that in the non-EA group (77.4% vs. 51.6%, *P =* 0.034) (Fig. 3). Multivariate logistic regression analyses indicated that serum YKL-40 levels significantly correlated with treatment responses defined by ΔACQ ≥0.5 or ΔFEV_1_ ≥ 12% (aOR = 0.82, 95% CI = [0.71, 0.96] adjusted for gender, age, BMI, and baseline FEV_1_% predicted and ICS dosage (Fig. 4).

## Discussion

To better understand the disease heterogeneity in asthma, patients have been grouped, or clustered, based on phenotypic and clinical profiles [40, 41]. This prospective cohort study in a real-world setting for the first time explored the relationship between serum YKL-40 and cellular inflammatory phenotypes of asthma. We showed that serum YKL-40 levels were significantly elevated in non-eosinophilic i.e. neutrophilic and paucigranulocytic asthma. Higher levels of YKL-40 were associated with increased T1 inflammatory biomarkers such as IL-1β and IL-6, indicating that YKL-40 may be useful in predicting exacerbation rates and responses to asthma treatment regimens. This suggests that serum YKL-40 should be further studied to further progress its utilisation for clinical relevance and asthma management.

Asthma is a heterogeneous disease with different phenotypes reflecting varied inflammatory patterns. In study I, we confirmed the different clinical characteristics of EA, NA and PGA phenotypes, and as in previous studies showed that subjects with NA were older, had longer asthma durations, and higher sputum IL-1β, IL-8 and TNF-α, but lower F_E_NO compared with EA [26–28]. Furthermore, our study found that serum YKL-40 levels were elevated in NA and PGA compared with EA, which is likely through elevated expression by various cell types including neutrophils and macrophages [42–44]. Recently published studies, which accord with our findings, have found this significant positive relationship between YKL-40 levels and blood neutrophil numbers in asthma [20, 45, 46]. The higher levels of blood neutrophils in patients with NA compared with EA, along with the correlation between YKL-40 and blood neutrophils (absolute numbers and percentage) in our study, strengthen the association of serum YKL-40 with NA. In addition, recent studies [47–49] found that YKL-40 levels in chronic obstructive pulmonary disease (COPD) and asthma–COPD overlap (ACO) were higher than asthma, which is consistent with increased neutrophilic inflammation in these diseases. However, similar to the recent findings by James et al. [22], our study did not observe relationship between serum YKL-40 and sputum neutrophils, which may be explained by different specimens although it indicated a moderate correlation between sputum and serum YKL-40 values [46].

Notably, we also showed that YKL-40 levels had significantly elevated in PGA, a form of NEA, and characterized by lower eosinophil and neutrophil but higher macrophage levels. The main cellular source of YKL-40 has been shown to be monocytes and macrophages [42, 50, 51]. The increased number of macrophages in PGA and the positive correlation between YKL-40 and blood monocytes (absolute numbers and percentage) in our study also support this observation, although we did not find relationship between YKL-40 and sputum macrophages. We found similar levels of YKL-40 between NA and PGA, which indicates that neutrophils would also be an important source, although less attention has been given to the underlying mechanisms that drive NEA [38]. Further, it has previously been reported that age is associated with NEA [28]. To exclude the effects of age on the relationship between YKL-40 and NEA, covariance analysis was used to show that the statistical between-group difference in YKL-40 remained significant after adjustment for age (*P* = 0.044). Although we did not measure YKL-40 levels in other matrices like plasma, sputum or urine, the difference of YKL-40 levels in serum across EA and NEA was more likely to result from the differential phenotypes of asthma rather than influences of matrices. Considering that matrices may have an effect on the expression of YKL-40, it needs further studies to explore YKL-40 levels in other matrices to differentiate NEA from EA.

There is a controversy with regard to the biological functions of YKL-40. In the past, YKL-40 was shown to be induced during T2 inflammation through an IL-13 dependent mechanism [51–53]. On the contrary, recent studies by Gomez and James suggested that YKL-40 and chitotriosidase were not T2-specific biomarkers in airway diseases [20, 22]. In our study we showed that YKL-40 expression positively correlated with non-T2 or T1 inflammation. Firstly, we found that serum YKL-40 levels were higher in NEA compared with EA after adjusting for age. Secondly, we demonstrated that YKL-40 negatively correlated with blood eosinophils and IgE, which are potential biomarkers of Th2-type inflammation [3, 21]. Thirdly, our study indicated that serum YKL-40 levels positively associated with non-T2 or T1 inflammation including blood neutrophils and IL-1β, which are consistent with previously published studies [4, 20]. IL-1β is a key mediator of inflammation, especially in the neutrophilic subtype of asthma [4, 28]. The cluster analysis by Gomez et al. showed that high serum YKL-40 clusters were characterised by airway neutrophils, where gene expression profiles showed distinct activation of the IL-1 pathway [20]. IL-6 has been shown to promote YKL-40 protein production, indicating the links between IL-6, inflammation and increased levels of YKL-40 [54]. Fourthly, Peters et al. found that the presence of higher levels of T2 mRNA expression such as IL-4, IL-5 and IL-13 in a cluster with low YKL-40 protein levels indicates that YKL-40 was primarily associated with non-T2 inflammatory pathways [55]. Taken together, although there were significant but weak correlations between serum YKL-40 and non-T2 biomarkers in our study, which provides further evidence that YKL-40 might be a non-T2 biomarkers, further investigations are required to discover its biological functions and validate its implications in asthma.

In our study, no association between YKL-40 and CCL22 was found, although the levels of CCL22 in the NA and PGA groups were significantly elevated compared with EA. There are controversies surrounding whether CCL20 belongs to Type 1 or Type 2 inflammation. Some studies indicate that CCL22 is a T2 inflammation biomarker [56, 57], but earlier study [58] found that CCL22 produced by microglia could regulate Th1-mediated central nervous system inflammation by facilitating the homing of Th2 and, possibly, regulatory T cells into the lesion site, which illustrates the complexities of the functions of CCL22. Our analysis of the four-week cohort dataset, found that asthma patients with NEA were relatively insensitive to asthma medication regimens, confirming clinical evidence that the NEA group generally responds poorly to corticosteroid treatment, which has little effect in reducing symptoms and restoring lung function [38, 59–61]. In molecular aspects, the elevated expression of non-T2 biomarkers such as IL-1β and IL-6 in NEA could play key roles in the lack of treatment responsiveness in NEA compared with EA [2]. Accordingly, this study indicated that YKL-40 may be an independent biomarker in predicting treatment responders, which is also supported by recently published studies [18, 22, 62]. Indeed, patients taking higher doses of ICS are also those with higher levels of YKL-40, suggesting that YKL-40 release is refractory to steroid treatment [18, 22, 62].

Asthma exacerbations, especially severe events, are periods of excess pathological and functional changes in the airways that have been proposed to induce airway remodeling [63–65]. Serum YKL-40 has been used as a surrogate marker for airway remodeling in asthma [16, 17]. Our study is the first in a prospective cohort design to find that serum YKL-40 is associated with increased risk of moderate to severe asthma exacerbations after adjusting for gender, age, BMI, baseline FEV_1_% predicted and ICS dose. We concluded that YKL-40 is a predictor of moderate-severe asthma exacerbation. However, we did not observe relationships between serum YKL-40 and severe exacerbations including hospitalizations and emergency department visits. This may be explained by several reasons. Firstly, in our study, a total of 21/109 (19.3%) patients who underwent moderate to severe exacerbations within the 12-month follow-up, the percentage seems unusually low, this may result from the fact that the majority of the asthmatic patients (87%) recruited had mild-to-moderate asthma in this cohort. Secondly, the relatively small sample size in our study may not be enough to assess the relationship of YKL-40 with severe exacerbations. However, Gomez et al. indicated that elevated serum YKL-40 levels were associated with two distinct clinical asthma phenotypes: one with irreversible airway obstruction and the other with severe exacerbations [20]. This issue therefore requires further investigations with a large cohort to confirm the association between serum YKL-40 and severe exacerbation risk.

Our study has considerable strengths. Firstly, the methods of assessment including lung function testing and sputum induction and had standard procedures according to the ASAN programme [29]. Secondly, sputum differential cell counts were performed by two well-trained laboratory researchers, and the results had near-perfect agreement (between 80.33–100.00% with Kappa from 0.722 to 1.00). This study also has several limitations that need to be addressed. Firstly, YKL-40 levels were measured only once during the one-year cohort study, which led that we could not explore whether serum YKL-40 level was an invariant characteristic despite asthma medication regimens. Others reported that the variability of serum YKL-40 over time was relatively low [22]. Secondly, our study lacked healthy subjects as the control group, although recently published studies found that serum YKL-40 levels in asthma patients were higher than healthy controls [22, 48]. Thirdly, we did not detect YKL-40 levels in different matrices such as plasma, sputum or urine, and analyze their relationships with inflammatory phenotypes of asthma because it was not the scope of this study. Fourthly, the percentage of severe asthma included in our study seemed unusually low, and we did not observe any correlation between YKL-40 and severe asthma. Fifthly, our study did not assess nucleotide variations in *CHI3L1* (gene encoding YKL-40) to explore the genotypes of YKL-40. Further experimental studies using accurate mouse models that recapitulate the critical features of severe asthma [28, 66] and exacerbations [67], and the use of genetically modified mice would help to understand the role of YKL-40 in pathogenesis and its use as a biomarker of specific disease features [23].

## Conclusions

In conclusion, we identified serum YKL-40 as a blood biomarker of NEA and demonstrated that it is associated with exacerbation and heterogeneity in response to one-month of asthma medication regimens that is in turn linked to inflammatory phenotypes. Serum YKL-40 positively correlated with non-T2 inflammatory signatures such as IL-1β and IL-6, which predicts insensitive responses to asthma treatment. This study suggests that serum YKL-40 is a novel promising biomarker of asthma inflammatory phenotypes with clinical relevance. Further studies are needed to assess whether monitoring YKL-40 levels could facilitate more accurate and successful clinical interventions.

## Additional file

Additional file 1: **Table S1.** Exacerbations within a 12-month follow-up in asthma patients grouped by YKL-40 levels. **Table S2.** Characteristics in the patients with asthma grouped by cellular inflammatory phenotypes in the study II. **Table S3.** Comparisons of characteristics in the patients with asthma between the studies I and II. (DOCX 32 kb)

## Abbreviations

ACO: Asthma–chronic obstructive pulmonary disease overlap
ACQ: Asthma control questionnaire
aOR: Adjusted odds ratio
AQLQ: Asthma quality of life questionnaire
ASAN: Australasian Severe Asthma Network
ATS: American Thoracic Society
BDP: Beclomethasone equivalents
BMI: Body mass index
CCL: Chemokine (C-C motif) ligand
CI: Confidence interval
COPD: Chronic obstructive pulmonary disease
DTT: Dithiothreitol
EA: Eosinophilic asthma
ED: Emergency department
ERS: European Respiratory Society
F_E_NO: Fractional exhaled nitric oxide
FEV_1_: Forced expiratory volume in 1 s
FVC: Forced vital capacity
GINA: Global Initiative for Asthma
ICS: Inhaled corticosteroid
ICU: Intensive care unit
IL: Interleukin
LABA: Long-acting beta-agonist
LTRA: Leukotriene receptor antagonist
NA: Neutrophilic asthma
NEA: Non- eosinophilic asthma
PGA: Paucigranulocytic asthma
Q: Quartile
SABA: Short-acting beta-agonist
SAWD: Severe Asthma Web-based Databas
SD: Standard deviation
TNF: Tumor necrosis factor
