# Supplementary material for: Chitinase-like protein YKL-40 correlates with inflammatory phenotypes, anti-asthma responsiveness and future exacerbations
Source: Respir Res. 2019 May 22;20:95. doi: 10.1186/s12931-019-1051-9 (PMC6530174; doi:10.1186/s12931-019-1051-9)
Supplement: Supplementary file 1 — Table S1. Exacerbations within a 12-month follow-up in asthma patients grouped by YKL-40 levels. Table S2. Characteristics in the patients with asthma grouped by cellular inflammatory phenotypes in the study II. Table S3. Comparisons of characteristics in the patients with asthma between the studies I and II. (DOCX 32 kb) [file 12931_2019_1051_MOESM1_ESM.docx]

Additional File 1

**Chitinase-like protein YKL-40 correlates with inflammatory phenotypes, anti-asthma responsiveness and future exacerbations**

Lei Liu, MD ^1, 2†^, Xin Zhang, MD, PhD ^1, 2, 3, 4†^, Ying Liu, MD ^1, 2^, Li Zhang, MD ^1, 2^, Jing Zheng, MD ^5^, Ji Wang, MD ^1, 2, 3^, Philip M. Hansbro, PhD ^6, 7, 8^, Lei Wang, MD ^1, 2^, Gang Wang, MD, PhD ^4*^, Alan Chen-Yu Hsu, PhD ^8^

Table S1. Exacerbations with a 12-month follow-up in asthma patients grouped by YKL-40 levels.

| Variables | YKL-40^low^ | YKL-40^high^ | t/χ^2^/Z | *P* value |
| --- | --- | --- | --- | --- |
| n | 57 | 52 |  |  |
| Moderate to severe exacerbations |  |  |  |  |
| Y, n (%) | 9 (15.8) | 12 (23.1) | 0.928 | 0.335 |
| Mean ± SD | 0.32 ± 0.83 | 0.50 ± 1.24 | -0.922 | 0.357 |
| Severe exacerbations |  |  |  |  |
| Y, n (%) | 7 (12.3) | 6 (11.5) | 0.014 | 0.905 |
| Mean ± SD | 0.21 ± 0.65 | 0.25 ± 1.03 | -0.135 | 0.893 |
| Non-planned visit |  |  |  |  |
| Y, n (%) | 5 (8.5) | 6 (11.5) | 0.229 | 0.632 |
| Mean ± SD | 0.16 ± 0.59 | 0.19 ± 0.60 | -0.470 | 0.638 |
| Emergency department visit |  |  |  |  |
| Y, % | 2 (3.5) | 4 (7.7) | 0.915 | 0.339 |
| Mean ± SD | 0.05 ± 0.29 | 0.29 ± 1.14 | -1.314 | 0.189 |
| Hospitalizations |  |  |  |  |
| Y, n (%) | 6 (10.5) | 3 (5.8) | 0.812 | 0.493 |
| Mean ± SD | 0.19 ± 0.64 | 0.08 ± 0.33 | -0.928 | 0.353 |
| ICU admissions, Y, n (%) | 1(1.8) | 0 (0.0) | 0.921 | 1.000 |

ICU=intensive care unit; SD=standard deviation; Y=yes.

Table S2. Study II: Characteristics of asthma patients grouped by cellular inflammatory phenotypes.

| Characteristics | EA | NEA | t/χ^2^/z | *P* Value |
| --- | --- | --- | --- | --- |
| n | 31 | 31 |  |  |
| Age, mean ± SD, yr | 40.4 ± 12.8 | 49.0 ± 16.2 | 2.305 | 0.025 |
| Gender, male n (%) | 11 (35.3) | 15 (48.4) | 11.060 | 0.303 |
| BMI, median (Q1, Q3), kg/m^2^ | 21.75 ± 3.69 | 23.46 ± 3.75 | 1.802 | 0.077 |
| Smoking, Current/Ex/Non | 5/3/22 | 5/6/20 | 1.079 | 0.583 |
| Age of asthma onset, mean ± SD, yr | 32.61 ± 14.07 | 36.65 ± 20.59 | 0.851 | 0.398 |
| Asthma duration, median (Q1, Q3), yr | 3.0 (1.0, 10.0) | 6.0 (2.0, 23.0) | -1.798 | 0.072 |
| ICS dose, BDP equivalent, median (Q1, Q3), μg/d | 400 (400, 800) | 400 (400, 400) | -0.654 | 0.513 |
| GINA steps 1/2/3/4/5, (n) | 0/0/31/1/0 | 0/0/26/5/0 | 2.952 | 0.195 |
| Medications, n (%) |  |  |  |  |
| ICS/LABA | 30 (96.8) | 30 (96.8) | 0.000 | 1.000 |
| LTRA | 27 (87.1) | 27 (87.1) | 0.000 | 1.000 |
| Theophylline | 7 (22.6) | 8 (25.8) | 0.088 | 0.767 |
| SABA | 2 (6.5) | 2 (6.5) | 0.000 | 1.000 |
| Spirometry, mean ± SD |  |  |  |  |
| FEV_1_, L | 2.23 ± 0.91 | 2.22 ± 0.92 | -0.068 | 0.946 |
| FVC, L | 3.23 ± 1.00 | 3.19 ± 0.92 | -0.159 | 0.874 |
| FEV_1_, % predicted | 71.27 ± 21.49 | 76.03 ± 20.71 | 0.888 | 0.378 |
| FVC, % predicted | 87.19 ± 15.25 | 91.71± 14.91 | 1.179 | 0.243 |
| FEV_1_/FVC, % | 68.11 ± 15.11 | 68.12 ± 14.43 | 0.823 | 0.442 |
| ACQ score, median (Q1, Q3) | 1.0 (0.17, 1.67) | 0.67 (0.0, 1.50) | 0.003 | 0.998 |
| AQLQ score, mean ± SD | 5.77 ± 0.76 | 5.82 ± 0.63 | 0.299 | 0.766 |
| F_E_NO, median (Q1, Q3), ppb | 55.0 (47.5, 80.5) | 19.0 (16.0,48.50) | -3.505 | 0.001 |
| IgE, median (Q1, Q3), IU/mL | 258.70 (178.76, 605.03) | 71.57(26.02, 444.70) | -3.310 | 0.002 |

ACQ= asthma control questionnaire; AQLQ=asthma quality of life questionnaire; BDP=beclomethasone equivalents; BMI=body mass index; EA=eosinophilic asthma; F_E_NO=fractional exhaled nitric oxide; FEV_1_=forced expiratory volume in 1 second; FVC=forced vital capacity; ICS=inhaled corticosteroid; ICS/LABA=inhaled corticosteroid with long-acting beta-agonist; LTRA=leukotriene receptor antagonist; NEA=non-eosinophilic asthma; SABA=short-acting beta-agonist; SD=standard deviation; Q=quartile.

Table S3. Comparisons of asthma patient characteristics between studies I and II.

| Characteristics | Study I | Study II | t/χ^2^/z | *P* Value |
| --- | --- | --- | --- | --- |
| n | 115 | 62 |  |  |
| Age, mean ± SD, yr | 45.9 ± 14.6 | 44.7 ± 15.1 | 0.527 | 0.599 |
| Gender, male n (%) | 48 (41.7) | 26 (41.9) | 0.001 | 0.980 |
| BMI, median (Q1, Q3), kg/m^2^ | 23.56 ± 4.29 | 22.61 ± 3.79 | 1.471 | 0.143 |
| Smoking, Current/Ex/Non | 14/21/80 | 10/9/42 | 0.811 | 0.667 |
| Age of asthma onset, mean ± SD, yr | 34.13 ± 17.54 | 34.63 ± 18.60 | -0.177 | 0.860 |
| Asthma duration, median (Q1, Q3), yr | 6.00 (2.00, 16.00) | 3.00 (2.00, 16.25) | -1.007 | 0.686 |
| ICS dose, BDP equivalent, median (Q1, Q3), μg/d | 400 (400, 400) | 400 (400, 400) | -0.404 | 0.314 |
| GINA steps 1/2/3/4/5, (n) | 0/0/105/10/0 | 0/0/56/6/0 | 0.047 | 0.828 |
| Medications, n (%) |  |  |  |  |
| ICS/LABA | 114 (99.1) | 60 (96.8) | 1.342 | 0.281 |
| LTRA | 102 (88.7) | 54 (87.1) | 0.098 | 0.754 |
| Theophyline | 20 (17.4) | 15 (24.2) | 1.175 | 0.278 |
| SABA | 4 (3.5) | 2 (6.5) | 0.825 | 0.364 |
| Spirometry, mean ± SD |  |  |  |  |
| FEV_1_, L | 2.25 ± 0.90 | 2.22 ± 0.91 | -0.068 | 0.946 |
| FVC, L | 3.20 ± 0.91 | 3.21 ± 0.96 | -0.064 | 0.949 |
| FEV_1_, % predicted | 74.65 ± 20.06 | 73.65 ± 21.06 | 0.310 | 0.757 |
| FVC, % predicted | 89.73 ± 14.14 | 89.45± 15.13 | 0.121 | 0.904 |
| FEV_1_/FVC, % | 68.59 ± 14.77 | 68.12 ± 14.73 | 0.202 | 0.840 |
| ACQ scores, median (Q1, Q3) | 0.50 (0.17, 1.33) | 0.92 (0.17, 1.54) | -1.143 | 0.253 |
| AQLQ scores, mean ± SD | 5.90 ± 0.71 | 5.79 ± 0.69 | 0.965 | 0.336 |
| F_E_NO, median (Q1, Q3), ppb | 27.50(17.00, 53.50) | 47.50 (17.0,74.25) | -1.466 | 0.145 |
| IgE, median (Q1, Q3), IU/mL | 171.52 (42.49, 380.88) | 201.41(42.02, 484.25) | -0.475 | 0.635 |
| YKL-40, ng/mL | 38.96 (27.48,61.68) | 38.49 (27.31,58.97) | 0.271 | 0.786 |

ACQ= asthma control questionnaire; AQLQ= asthma quality of life questionnaire; BDP=beclomethasone equivalents; BMI=body mass index; F_E_NO=fractional exhaled nitric oxide; FEV_1_=forced expiratory volume in 1 second; FVC=forced vital capacity; ICS=inhaled corticosteroid; ICS/LABA=inhaled corticosteroid with long-acting beta-agonist; LTRA=leukotriene receptor antagonist; SABA=short-acting beta-agonist; SD=standard deviation; Q=quartile.
